# An Assessment of the Potential Impact of Fortification of Staples and Condiments on Micronutrient Intake of Young Children and Women of Reproductive Age in Bangladesh

**DOI:** 10.3390/nu8090541

**Published:** 2016-09-02

**Authors:** Magali Leyvraz, Arnaud Laillou, Sabuktagin Rahman, Tahmeed Ahmed, Ahmed Shafiqur Rahman, Nurul Alam, Santhia Ireen, Dora Panagides

**Affiliations:** 1 Global Alliance for Improved Nutrition (GAIN), Geneva 1211, Switzerland; dpanagides@gainhealth.org; 2 United Nations Children’s Fund (UNICEF), Phnom Penh 12201, Cambodia; alaillou@unicef.org; 3 International Centre for Diarrhoeal Disease Research (ICDDR), Dhaka 1000, Bangladesh; sabuktagin@icddrb.org (S.R.); tahmeed@icddrb.org (T.A.); ashafiq@icddrb.org (A.S.R.); nalam@icddrb.org (N.A.); santhia@icddrb.org (S.I.)

**Keywords:** micronutrients, food fortification, rice, wheat flour, oil, women of reproductive age, young children, Bangladesh

## Abstract

Bangladesh has experienced rapid economic growth and achieved major health improvements in the past decade, but malnutrition rates remain high. A nationally representative study conducted in 2011 assessed the dietary habits of 841 children 24–59 months old, 1428 children 6–14 years old, and 1412 nonpregnant, nonlactating women. The study’s objective was to assess dietary intakes of key micronutrients and the consumption pattern of potentially fortifiable foods, and then to model the potential impact of the fortification of key staple foods. The current intakes of several micronutrients—namely, iron, zinc, folate, vitamin A, and vitamin B12—were found to be insufficient to meet the needs of Bangladesh’s children and women. The fortification of rice with iron and zinc and edible oil with vitamin A has the potential to fill a significant part of the nutrient gap, as these are consumed widely and in significant amounts. Wheat flour and sugar are not as promising food vehicles in the Bangladeshi context, as they were consumed by a smaller portion of the population and in smaller amounts. In conclusion, fortification of rice and oil is recommended to address the large gap in micronutrient intakes.

## 1. Introduction

Malnutrition is a widespread public health problem, especially in developing countries. According to The State of Food Insecurity in the World [1], 870 million people suffer from undernourishment and around two billion people are affected by micronutrient deficiencies. The consequences of malnutrition are multiple, ranging from reduced physical capacity and impaired intellectual development to increased morbidity and mortality. It has been estimated that 3.1 million children die from malnutrition every year [2].

Bangladesh is a developing country that has experienced rapid economic growth during the last 20 years, with an annual gross domestic product growth rate of 6.1% [3]. Simultaneously, poverty and malnutrition rates have been significantly reduced [1] and Bangladesh is considered to be on track to achieve many of its Millennium Development Goal targets [4]. However, poor nutritional status is still a major problem. The 2011 Bangladesh Demographic and Health Survey [4] estimated that 41% of the children under 5 years of age are stunted, 16% are wasted, and 36% are underweight. Moreover, the December 2011 National Micronutrients Status Survey [5] found a high prevalence of micronutrient deficiencies among preschool children and nonlactating, nonpregnant women: 21% of the children and 5% of the women were vitamin A deficient, 11% of the children and 7% of the women were iron deficient, and 45% of the children and 57% of the women were zinc deficient. In addition, 9% and 6% of the women were folate and vitamin B12 deficient, respectively.

There are a number of factors that contribute to the high prevalence of micronutrient deficiencies, including low consumption of animal-source foods, poor bioavailability of micronutrients from plant sources, diseases and infections, food shortages, and, most importantly, a limited dietary diversity [6]. Energy intake and dietary diversity are significantly associated with micronutrient intake in women and children in Bangladesh [7,8]. A study conducted in rural Bangladesh showed that only 44% of children between 2 and 4 years old and 30% of nonlactating women had adequate micronutrient intakes [7]. All respondents consumed starchy staples. However, micronutrient-rich foods were consumed by a much smaller segment of the population: only 69% of the children and 84% of the women consumed meat products; 46% and 30%, respectively, consumed eggs; 48% and 22% consumed dairy products; 71% and 64% consumed legumes and nuts; 46% and 71% consumed vitamin A-rich green leafy vegetables; and 48% and 52% consumed other vitamin A-rich fruits and vegetables. Therefore, to improve the population’s micronutrient intake, increasing the micronutrient content of the widely consumed staple foods through fortification could be a promising intervention: the majority of the population can be reached, and reached fairly quickly.

Fortification is widely recognized as being one of the most cost-effective strategies to increase regular consumption of micronutrients in a population. However, to be successful, any fortification program needs to take into account a number of parameters [6]. First, the spread and severity of micronutrient deficiencies need to be assessed and the gap in micronutrient intake evaluated. Then, the consumption patterns need to be investigated to identify potential vehicles for fortification. Finally, based on these findings, as well as on technological and cost parameters, an optimal fortification strategy can be designed.

The objective of this study was to assess dietary intakes of key micronutrients and the consumption pattern of potentially fortifiable foods, and then, based on this information, to model the potential impact of fortification of key staple foods and to assess variation in all of these foods by regions of the country. The findings of this study aim at informing the design of a fortification strategy in Bangladesh.

## 2. Experimental Section

### 2.1. Study Design

The data presented in this paper were collected as part of a large cross-sectional nationally representative survey, the National Micronutrients Status Survey December 2011 [5], that was conducted between October and December 2011 in Bangladesh. Information on micronutrient deficiencies, food consumption, socioeconomic parameters, knowledge, and practices was collected.

### 2.2. Population Sampling

The National Micronutrients Status Survey December 2011 [5] used a three-stage sampling to survey preschool-aged children (pre-SAC) 6–59 months old, school-aged children (SAC) 6–14 years old, and nonpregnant, nonlactating women of reproductive age (NPNL) 15–49 years old. The targeted populations were selected from 3 strata: rural, urban, and urban slums. In the first stage, 50 primary sampling units (PSUs) per stratum (i.e., 150 PSUs in total) were randomly selected from the list of the 15,000 primary sampling units that was used for the Bangladesh Multiple Indicator Cluster Survey (MICS 2009). In the second stage, a segment containing 50 households was selected per PSU, from which 20 households were randomly selected to be included in the survey. In the third stage, approximately 8 pre-SAC, 10 SAC, and 10 NPNL were selected among the 20 households in each PSU. In order to minimize nonresponse, nonresponders were substituted with other eligible respondents randomly selected from the 20 households selected in the second stage until the required sample sizes for each population group were achieved.

### 2.3. Food Consumption Analysis

In total, consumption data were gathered from 841 pre-SAC 24–59 months, 1428 SAC, and 1412 NPNL between October and December 2011. The surveyed population included pre-SAC 6–23 months old, however information on their consumption was not assessed, as they were expected to consume limited amounts of the food vehicles and therefore to benefit from fortification of staples to a limited extent only.

To assess food consumption and nutrient intake of NPNL, SAC, and pre-SAC, individual 7-day semiquantitative food frequency questionnaires were used to interview NPNL, SAC, and the caregivers of pre-SAC between 24 and 59 months, taking into consideration commonly consumed foods, with special attention to vitamin A-, iron-, and zinc-rich foods. The only food that was not assessed by the semiquantitative food frequency questionnaire was oil, which was assessed at the household level. Food photographs indicating the serving size and amount in grams (g) were used to assess the quantity of consumption. Raw food weight was calculated by using appropriate conversion factors [9]. Micronutrient intakes (of iron, zinc, and vitamin A) were calculated per 100 g of raw food consumed using the most updated Bangladeshi Food Composition Table [10]. To transform the vitamin A intakes into a retinol equivalent (RE), 1 µg of vitamin A from animal sources was deemed equal to 1 RE, and 12 µg of vitamin A from plant sources was deemed equal to 1 RE [11].

The wheat flour products consumed by the households were separated into two categories: (1) bread, which was made industrially and bought already baked by the household and was made mainly from Maida flour; and (2) wheat flour, which was bought by the household and used to make foods within the household (e.g., chapatis or paratas) and consisted mainly of *atta* flour. The sugar consumption was estimated from the amount of sugar added to the foods or drinks consumed.

For vegetable oil, the data on household monthly expenses were gathered and an average consumption was estimated for each household. Consumption estimates were extrapolated from the average per-person consumption reported in the national surveys by applying adult equivalent units (AEUs), recommended by the Food and Agriculture Organization of the United Nations (FAO), by a factor of 0.51 for children 1–3 years old, 0.71 for children 4–6 years old, 0.78 for children between 7 and 10 years old, 0.98 for boys and 0.86 for girls between 11 and 14 years, and 0.86 for women of reproductive age (14–50 years of age) [12].

### 2.4. Socioeconomic Status

Socioeconomic status (SES) was calculated [13], using the Demographic Health Statistic Wealth Index to divide households surveyed into five socioeconomic quintiles: the “extreme poor” (category 1), the “poor” (category 2), the “intermediate” (category 3), the “wealthy” (category 4), and the “wealthiest” (category 5). As a measure of SES, we used an index that was created with information on household characteristics. Variables included land (homestead, land under cultivation); construction materials of the walls, roof, and floor of the house; ownership of household assets (electricity, radio, television, mobile phone, land phone, chair, watch, table, cupboard, rickshaw, van, animal-drawn cart, refrigerator, motor boat); and type of toilet facility. The categories for construction materials of the roof and walls were tin, brick–cement (pacca), and “other,” while those of floors were brick–cement (pacca), mud, and “other.” For household assets, each item belonged to 1 of 2 categories: “owned” or “not owned” by the household. Principal component analysis (PCA) was used to create the index. A weight was attached to each item from the first principal component.

### 2.5. Fortification Levels

Fortificants and fortification levels of selected foods are presented in Table 1. These levels were set according to the current and proposed Bangladesh standards for vitamin A in oil. The levels of fortification of wheat flour, rice, and sugar were set according to international recommendations: wheat flour fortification levels were determined based on the assumption of an adult’s equivalent consumption under 75 g/day [14], rice fortification levels were based on the assumed adult consumption of more than 300 g/day [15] (according to the World Health Organization (WHO)/Global Alliance for Improved Nutrition (GAIN) Technical Considerations for Rice Fortification in Public Health), and the fortification level of sugar was set as the average level of all current sugar fortification programs [6]. For calculation purposes, folic acid was transformed into a dietary folate equivalent (DFE): 1 µg of folic acid was equaled to 0.6 DFE and 1 µg food folate was equaled to 1 DFE.

### 2.6. Data Management and Statistical Analysis

WHO recommended nutrient intake (RNI) [16] was used to assess the potential contribution of dietary intakes of rice, wheat flour, sugar, and vegetable oil to micronutrient daily requirements. To calculate micronutrient intakes, the database of consumed food items was linked to food composition data from the Bangladesh Food Composition Database [10]. The upper micronutrient intake levels were calculated based on the upper 95% confidence interval (CI) of micronutrients and the upper 95% food vehicle consumption.

Data entry, including quality checks, as well as data management and analysis, was performed using SPSS software version 19. The mean consumptions among pre-SAC, SAC, and NPNL consuming rice, bread, vegetable oil, sugar, and wheat flour (excluding nonconsumers) were analyzed by one-way analysis of variance (ANOVA) (since the distribution of consumption was asymmetric) and multiple comparisons to compare strata, geographic areas, and socioeconomic status. To estimate the additional intake of the micronutrients under consideration for the fortification strategy, the mean consumption of nutrients from the fortifiable food (rice, wheat flour, vegetable oil, bread, and sugar) by age groups were calculated by multiplying the level of fortificant (including the losses during processing and storage: 5% for iron and zinc, 50% for folic acid, and 30% for vitamin A and vitamin B12 [6]) with the consumption of the different food vehicles. The losses in bread and wheat flour due to processing were assumed to be the same at the bakery and at the home. Iron bioavailability was estimated to be low (5%) for diets rich in cereals but including high sources of vitamin C [6]. A low absorption of zinc (15%) was also assumed, considering the diet in Bangladesh is rich in phytate and poor in animal-source food [6].

### 2.7. Ethical Issues

Ethical approval for this study was obtained from the Institutional Review Board (IRB) of the International Centre for Diarrhoeal Disease Research, Bangladesh (ICDDR,B) (Reference number: PR#11018). Under the IRB, the Research Review Committee provided approval on the technical aspects of the protocol, which was followed by the Ethical Review Committee giving approval for the study. The participants and/or the guardians of the participants in the study provided their written informed consent.

## 3. Results

**Figure 1:**
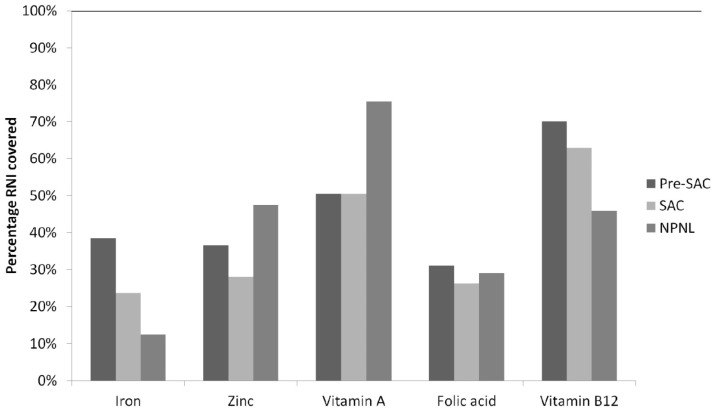
Percentage of iron, zinc, vitamin A, folate, and vitamin B12 recommended nutrient intake (RNI) covered by the diet per population group.

The consumption patterns of 841 pre-SAC, 1428 SAC, and 1412 NPNL were analyzed. The micronutrient intake estimates for five micronutrients are reported in Table 2 and Figure 1. For pre-SAC, 38.5%, 36.6%, 50.5%, 31.0%, and 70.0% of the requirements in iron, zinc, vitamin A, folic acid, and vitamin B12, respectively, were covered by the diet. The iron and zinc intakes varied significantly depending on the district of residency (*p* < 0.01 for iron and *p* < 0.05 for zinc). Zinc intake was higher in higher income quintiles (*p* < 0.001). There were no significant differences in folic acid intake between areas of residency, districts, nor socioeconomic strata. Vitamin B12 intake was significantly lower in pre-SAC from lower SES than from higher SES (*p* < 0.05).

For SAC, mean iron, zinc, vitamin A, folic acid, and vitamin B12 intakes in the diet covered 23.7%, 28.0%, 50.5%, 26.2%, and 62.9% of the needs, respectively. Iron intake was correlated with the district of residency (*p* < 0.01) and zinc and vitamin A intakes with SES (*p* < 0.001 and *p* < 0.001). Intake of folic acid was lowest in rural areas and highest in slums (*p* < 0.001). Consumption of vitamin B12 was proportional with socioeconomic strata (*p* < 0.001).

The diet of NPNL covered 12.5% of their requirements in iron, 47.4% in zinc, 75.4% in vitamin A, 29.0% in folic acid, and 45.6% in vitamin B12. Iron and zinc intakes were the highest in women from higher socioeconomic strata (*p* < 0.01 and *p* < 0.001, respectively). Vitamin A intake was correlated with the district of residency (*p* < 0.001) and the SES (*p* < 0.01). Intakes of folic acid and vitamin B12 were significantly (*p* < 0.01) lower in rural and lower-income households and higher in slum areas and higher-income households. Vitamin B12 consumption was lower in poorer households than in wealthier households (*p* < 0.001).

The consumption of the main fortifiable foods (i.e., rice, bread, wheat flour, sugar, and oil) was assessed for each population group. The percentages of pre-SAC, SAC, and NPNL consuming rice, bread, wheat flour, and sugar are shown in Table 3. Oil was consumed in 99.8% of the households.

The mean consumption of rice, wheat flour, bread, sugar, and oil by population group is shown in Table 4. Rice consumption was influenced by the place of living and the socioeconomic strata. Rice consumption was the highest in rural regions and the lowest in slums in all three population groups (*p* < 0.01, *p* < 0.05, and *p* < 0.001, respectively). The pre-SAC from the lower-income families consumed more rice than the pre-SAC from the higher-income families (*p* < 0.001). There were no significant differences in bread consumption between regions. However, wealth had an influence on the bread consumption of pre-SAC (*p* < 0.01): pre-SAC from wealthier families consumed more bread. SAC consumed significantly higher amounts of wheat flour in slums and urban areas than in rural areas (*p* < 0.001). Pre-SAC in slums tended to consume higher amounts of wheat flour and smaller amounts in rural areas (*p* = 0.05). SAC and NPNL from higher-income quintiles consumed more wheat than lower-income quintiles (*p* < 0.001). There was a significant difference in wheat flour consumption between the divisions: wheat flour consumption was the lowest in Dhaka and in Khulna and the highest in Chittagong and Rajshahi (*p* < 0.001). NPNL living in slums and urban areas consumed higher amounts of sugar than NPNL living in rural areas. Sugar was consumed in higher amounts by SAC and NPNL from higher-income quintiles (both *p* < 0.001). Consumption also significantly differed between divisions for SAC and NPNL (both *p* < 0.001). SAC in Barishal consumed the most sugar, while children in Sylhet consumed the least. In Rajshahi, NPNL consumed the most sugar and NPNL in Dhaka consumed the least sugar. The average daily oil consumption was 26.4 g per capita. Oil consumption was higher in urban and slum areas than in rural areas (*p* < 0.001). The highest oil consumption was found in Chittagong and the lowest in Barishal (*p* < 0.001).

**Figure 2:**
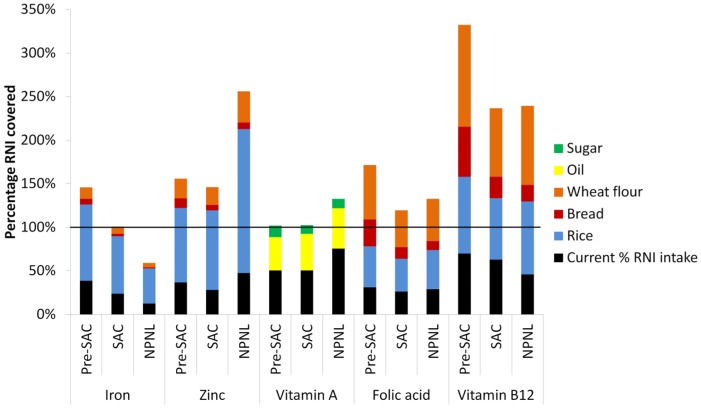
Estimated total RNI intake from current diet and fortified rice, bread, wheat flour, sugar, and oil.

**Figure 3:**
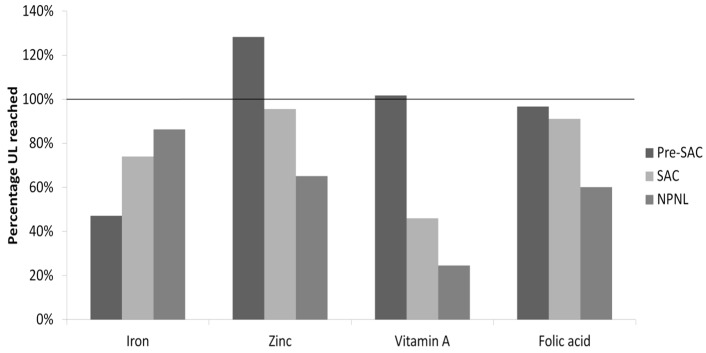
Estimated percentage of upper limit reached from multiple food fortification (based on the upper 95% consumption level and micronutrient intake).

Based on mean level of consumption and recommended fortification levels, the additional intakes of iron, zinc, folic acid, and vitamin B12 (Table 5), and vitamin A (Table 6) in case of fortification of all five foods were calculated, and the total percentage of RNI covered is reported in Figure 2. Moreover, based on the upper 95% consumption level and micronutrient intake, the estimated percentage of upper limit (UL) reached from multiple food fortification is reported in Figure 3. The estimated maximum total iron intake after fortification of rice, bread, and wheat flour ranges between 18.9 mg/day and 38.9 mg/day, depending on the target population, and stays below the upper level (40 mg/day) set by the WHO [6]. Zinc intakes could rise to 15.4 mg/day for pre-SAC, 22.0 mg/day for SAC, and 29.3 mg/day for NPNL, which is above the WHO upper level for pre-SAC (7–12 mg/day) and within the upper limits for SAC (12–23 mg/day), below the upper limits for NPNL (45 mg/day). The total maximum intakes of folic acid remain below the age-group-specific ULs (i.e., 300–400 µg/day for pre-SAC; 400–600 µg/day for SAC; and 1000 µg/day for adults). The total maximum intakes of folic acid also stay below the age-group-specific ULs (i.e., 300–400 µg/day for pre-SAC; 400–600 µg/day for SAC; and 1000 µg/day for adults). The additional vitamin A intake would be far below the upper levels for NPNL (3000 RE/day) and SAC (900–1700 RE/day); however, it is slightly too high for pre-SAC (700 RE/day).

## 4. Discussion

According to the data collected from the food frequency questionnaires, iron, zinc, and vitamin A intakes were found to be lower than the requirements, highlighting the need for nutrition interventions. These findings are especially alarming since they concern population groups for whom it is critical to have an adequate nutrient intake. In fact, nutritional deficiencies during infancy and childhood can have serious and irreversible consequences that last until adulthood [17,18]. Moreover, micronutrient needs during pregnancy strongly increase. Therefore, it is essential that women build their micronutrient stores, especially of iron and folic acid, to ensure optimal nutritional status during pregnancy and a healthy infant after delivery [19,20]. Fortification of staple food would also benefit women after pregnancy and during lactation, especially since the WHO no longer recommends vitamin A supplementation postpartum [21].

Zinc intakes in individuals from higher socioeconomic strata were higher than those in the lower strata. Women from lower socioeconomic strata tended to have lower iron intakes. This finding highlights the need to design an intervention that reaches the poorest segments of the population, especially for zinc and iron. These minerals are contained in a number of foods and in highest amounts in animal-source products. However, their absorption can be significantly inhibited by certain dietary components, such as phytic acid and polyphenols [22,23]. Unfortunately, diets among poorer Bangladeshi households are less diverse, with higher amounts of staples, which are rich in iron and zinc absorption inhibitors, and with lower amounts of animal-source foods, which are rich in highly bioavailable iron and zinc [24].

Based on the consumption patterns, rice is the most suitable vehicle for fortification: it is consumed regularly by the whole population, in very large and constant amounts. In fact, rice fortification could provide between 10.4 mg/day and 23.9 mg/day of additional iron, or an additional 40.5%–87.5% of the population’s RNI in iron. Rice fortification alone would be sufficient to reduce the prevalence of iron deficiency in the population. In fact, efficacy studies have shown that daily iron intakes from rice of between 13 mg and 20 mg can reduce iron deficiency and iron deficiency anemia [25,26,27]. Rice fortification could also provide between 7.5 mg/day and 17.1 mg/day of zinc, or 85.4%–165.4% of the zinc RNI. Since rice fortification with zinc has not yet been proven efficacious, additional studies are warranted to evaluate the efficacy of this intervention. Moreover, the risks of providing amounts above or close to the UL for pre-SAC and SAC should be assessed. Rice fortification would provide additional amounts of folate and vitamin B12, which are also insufficient in the diets of the Bangladeshi. However, there are a number of challenges with rice fortification that still need to be solved to ensure the success and sustainability of a rice fortification program [28].

As with rice, oil is consumed by almost all households. Oil could provide 38.3%–46.5% of the population’s vitamin A requirements, which is above the 15% minimum recommended by the International Vitamin A Consultative Group [29]. However, since this study estimated oil consumption at the household level, it is difficult to estimate the actual consumption and therefore an exact RNI contribution for each population group.

In comparison to rice and oil, the number of people consuming bread, wheat, and sugar, and the amounts consumed, are much lower. However, fortification of these food vehicles would significantly contribute to filling the gaps in current micronutrient intakes, especially if it is implemented alongside fortification of other foods (e.g., rice and oil) and in combination with other interventions, such as dietary diversification and supplementation. The fortification of wheat flour would provide additional amounts of iron and zinc, but also (and mostly) folate and vitamin B12. *Maida* flour fortification would provide more micronutrients to pre-SAC and SAC than to NPNL. *Atta* flour fortification would provide more micronutrients to children living in slums and urban areas, whereas rice fortification would provide more to the rural populations. Sugar fortification with vitamin A would complement the vitamin A intakes provided by the diet and by fortified oil and would result in sufficient intakes of vitamin A among all three population age groups. However, the percentage of respondents who reported consuming sugar and the amounts consumed reported were much lower than for oil. The fortification of sugar would not benefit all population groups equally: it would benefit mostly a small segment of young children from higher socioeconomic strata.

According to the results of this analysis, the simultaneous fortification of wheat flour and rice with iron would be enough to cover the needs of iron, zinc, folic acid, and vitamin B12 for pre-SAC, SAC, and NPNL, at the exception of the iron needs of NPNL. Therefore, fortification efforts need to be combined with other strategies to prevent iron deficiency. The WHO recommends three food-based strategies to fight iron deficiency: dietary diversification, food fortification, and iron supplementation. To maximize the reduction of iron deficiency prevalence among women in Bangladesh, food fortification could be combined with iron supplementation for iron-deficient women.

The iron intakes might have been underestimated in our study. In fact, iron intakes were estimated based on food consumption and did not consider iron provided by groundwater. A study has shown that groundwater could provide significant amounts of iron to women in rural areas of Bangladesh [30]. Therefore, for women living in rural areas of Bangladesh, the levels fortification should be defined taking into consideration of the amounts of iron taken from groundwater, and consequently the decision to fortify both wheat flour and rice with iron or only a single food vehicle should be based on the actual gap between iron intakes from all sources and needs.

The risk of excess intake from the fortification of multiple food vehicles is relatively low, especially given the fact that it is rare for an individual to consume all fortified products in one day. The only areas of concern would be the vitamin A intake in pre-SAC and zinc intake in pre-SAC and SAC. Some children might consume amounts of vitamin A that are close to the upper levels set for that age group. In addition, these same children have a high chance of receiving high-dose vitamin A supplements. Should the simultaneous of fortification of multiple vehicles be implemented, the risk of exceeding UL for the two micronutrients should be carefully assessed.

There are four major limitations to this study. The first one is that the consumption data were assessed using a seven-day semiquantitative food frequency questionnaire. Micronutrient intakes might have been slightly over- or underestimated [31,32]. The seven-day semiquantitative food frequency questionnaire has been validated against 24 h recalls in China [33] and seven-day dietary diary in Bangladesh [30]. However, in order to validate the findings of this study, a comparison with a subsample of repeated 24 h recalls would have been recommended. The second limitation is that under- and over-reporting were not taken into consideration. A study collected information on diet by a seven-day semiquantitative food frequency questionnaire and a seven-day diary reported that meat consumption was under-reported and fruit consumption over-reported [31]. The third limitation is that individual oil consumption was estimated based on household purchase and conversion rates. To get a realistic estimate of intrahousehold oil consumption, oil and oil-containing dishes should be assessed individually. The fourth limitation is that the consumption of the food vehicles by children between 6 and 23 months old was not assessed. Although the population in this age group is expected to consume limited amounts of these food vehicles, fortification of staples can also contribute to increasing the micronutrient intakes of this high-risk population. Specific interventions, in addition to fortification of staples, need to be developed to improve the micronutrient intakes of this high-risk population group, such as fortified supplementation, home fortification, and fortified complementary foods.

## 5. Conclusions

In conclusion, rice and oil consumption were high, constant, and widespread across Bangladesh and therefore are the most promising food vehicles for fortification. Fortification of these two food vehicles could significantly increase the micronutrient intakes of most population groups. In order to design an optimal fortification strategy, these findings need to be complemented with a comprehensive industry and market assessment and the fortification levels adapted based on cost, technological and sensory parameters.

## Figures and Tables

**Table 1:** Standards for an integrated fortification strategy.

Fortificant	Standard
Rice	70 mg/kg of iron as micronized ferric pyrophosphate, 50 mg/kg of zinc, 1 mg/kg of folic acid, and 0.008 mg/kg of vitamin B12
Wheat flour	40 mg/kg of iron as NaFeEDTA, 50 mg/kg of zinc, 5 mg/kg of folic acid, and 0.04 mg/kg of vitamin B12
Sugar	10 parts per million (ppm) of retinyl palmitate
Vegetable oil	10–15 ppm of retinyl palmitate

**Table 2:** Mean (95% confidence interval, CI) micronutrient intake by population group per capita.

Micronutrients	Preschool-Aged Children (Pre-SAC)	School-Aged Children (SAC)	Nonpregnant Nonlactating Women (NPNL)
Iron (mg/day)	4.6 (3.2–5.0)	6.2 (5.6–7.0)	7.4 (7.0–8.0)
Zinc (mg/day)	3.2 (2.8–4.0)	3.8 (3.4–4.0)	4.9 (4.3–5.0)
Vitamin A (RE/day)	210.6 (75.0–468.4)	277.9 (91.7–620.1)	385.8 (127.5–780.1)
Folic acid (µg/day)	51.7 (47.7–55.7)	90.3 (85.6–95.7)	116.0 (110.4–121.9)
Vitamin B12 (µg/day)	0.7 (0.6–0.8)	1.3 (1.2–1.4)	1.1 (1.1–1.2)

**Table 3:** Percentage of people consuming rice, bread, wheat flour, and sugar by population group.

Fortifiable Foods	Pre-SAC	SAC	NPNL
Rice	99.3%	100.0%	100.0%
Bread	33.5%	28.0%	10.5%
Wheat flour	76.2%	66.5%	41.9%
Sugar	41.1%	36.7%	30.3%

**Table 4:** Mean (95% CI) consumption of rice, bread, wheat flour, sugar, and oil by consuming population groups (g/capita/day).

Fortifiable Foods	Pre-SAC	*n*	SAC	*n*	NPNL	*n*
Rice	157.1 (147.6–166.4)	841	260.6 (233.1–288.1)	1428	359.5 (318.6–400.4)	1412
Bread	20.5 (16.3–24.7)	284	18.0 (14.4–21.6)	400	16.2 (12.7–19.6)	207
Wheat flour	41.8 (35.0–48.6)	646	58.2 (47.6–68.8)	1050	77.9 (63.8–92.0)	907
Sugar	7.8 (5.8–9.8)	348	7.9 (6.3–9.5)	530	7.8 (5.3–10.2)	485
Oil	15.2 (13.8–16.6)	2000	22.0 (19.9–24.0)	2000	22.7 (20.6–24.8)	2000

**Table 5:** Estimated daily contribution from fortified rice, bread, and wheat flour (using mean amounts consumed per day by each population group, and proposed fortification levels).

	Iron (mg/Day)	Zinc (mg/Day)	Folic Acid (µg/Day)	Vitamin B12 (µg/Day)
Rice				
Pre-SAC	10.4	7.46	78.6	0.9
SAC	17.3	12.4	130.3	1.5
NPNL	23.9	17.1	179.8	2.0
Bread				
Pre-SAC	0.8	1.0	51.3	0.6
SAC	0.7	0.9	45.0	0.5
NPNL	0.6	0.8	40.5	0.5
Wheat flour				
Pre-SAC	1.6	2.0	104.5	1.2
SAC	2.2	2.8	145.5	1.6
NPNL	3.0	3.7	194.8	2.2

**Table 6:** Estimated daily contribution to vitamin A intake from fortified sugar and oil (using mean amounts consumed per day by each population group, and proposed fortification levels).

Population Groups	Vitamin A (µg Retinol Equivalent/Day)
	Sugar
Pre-SAC	54.6
SAC	55.3
NPNL	54.6
	**Oil**
Pre-SAC	159.6
SAC	230.9
NPNL	238.0
